# Knowledge, attitude, prevention practice, and associated factors toward COVID-19 among preparatory school students in Southwest Ethiopia, 2021

**DOI:** 10.1371/journal.pone.0262907

**Published:** 2022-01-24

**Authors:** Mohammed Yesuf, Mehd Abdu

**Affiliations:** Department of Nursing, College of Medicine and Health Science, Mizan-Tepi University, Mizan-Teferi, Ethiopia; Shahid Beheshti University of Medical Sciences, ISLAMIC REPUBLIC OF IRAN

## Abstract

**Introduction:**

As of February 2021 COVID-19 report in 57 African countries, there were 3,761,512 confirmed cases and 98,088 deaths. Ethiopia reported the highest number of cases in East Africa with a total of 147,092 cases and 2,194 deaths. Over 1.5 billion students from 195 countries across the world separated from school as a consequence of the closure of schools related to the pandemic. This study aimed to determine the level of knowledge, attitude, prevention practices, and determinant factors regarding COVID-19 among preparatory school students in southwest Ethiopia.

**Methods:**

An institution-based cross-sectional study design was used for 422 samples. Each respondent was selected using simple random sampling. Data were collected using a self-administered questionnaire. The collected data were entered and analyzed using Statistical Package for social science software version 25.0. Multivariable binary logistic regression was used to identify factors that were significantly associated with the practice of COVID-19 prevention.

**Results:**

The response rate in this study was 96.2%. A higher proportion of the respondents were female (53.9%), Bench (43.6%), and protestant (47.3%). The level of good knowledge, positive attitude, and good practice were 81.8%, 70.9%, and 47.0% respectively. Using social media [AOR: 1.801, 95% CI: 1.005, 3.226], watching television [AOR: 1.884 95% CI: 1.093, 3.247], being knowledgeable [AOR: 5.173 95% CI: 2.276, 11.755], and having a positive attitude [AOR: 4.300 95% CI: 2.351, 7.868] were positively associated with COVID-19 prevention practice.

**Conclusion:**

Despite the high level of knowledge and a moderate level of positive attitude, the practice of COVID-19 prevention measures was low. Using social media, watching television, being knowledgeable, and having positive attitudes towards COVID-19 increases the tendency to practice COVID-19 prevention measures. School directors and teachers should strictly monitor students for their adherence to COVID-19 prevention measures as directed by the local and national health care departments.

## Introduction

In Wuhan city of China, a group of pneumonia cases of unidentified etiology was reported on December 31, 2019. The China CDC (Center for Disease Control and Prevention) reported novel coronavirus as the etiologic agent of this pandemic on January 9, 2020. The outbreak originated in the edible aquatic life market in Wuhan, China. However, further investigation found that a few individuals became infected without a history of visiting the market for edible aquatic life. The disease state associated with this virus is termed coronavirus disease 2019 (COVID-19). The World health organization director-general declared the COVID-19 pandemic in March 11 2020 [1, 2].

The word COVID-19 stands for Coronavirus Disease 2019. Coronaviruses are a group of microorganisms belonging to the *Coronaviridae* family. It infects both animals and humans. Most human coronavirus infections can cause mild common cold-like symptoms and others may cause serious illnesses like SARS (Severe Acute Respiratory Syndrome) and MERS (Middle East Respiratory Syndrome) [3].

As of February 14 2021, COVID-19 affected 235 countries which resulting in a total of 106,477,025 cases and 2,361,848 deaths worldwide. In Africa, 57 countries reported the case (3,761,512 cases and 98,088 deaths across the continent). Ethiopia reported the highest number of cases in East Africa with a total of 147,092 confirmed cases and 2,194 deaths as of 14^th^ February [4].

The main mechanism for person-to-person transmission of COVID-19 is through the droplets of saliva or discharge from the nose while the infected person sneezes or coughs. It can also be spread via direct or indirect contact with an infected person. Respiratory droplets or aerosols can penetrate the lungs via inhalation through the nose or mouth [1]. The spread of the organism can be halted by proper handwashing, maintaining physical distancing, avoiding touching faces with the hand, and covering mouth and nose with a face mask [5, 6].

The hazard of the spread of infectious agents is high in social organizations with high-risk populations. The influence of transmission of the organism in such social organizations can be halted by the proper application of infection prevention and monitoring strategies [2].

To minimize the transmission rate of infectious agents, educational organizations in many countries around the world temporarily suspended face-to-face instruction. Schools in many countries have closed since early March 2020 [7]. Over 1.5 billion students from 195 countries across the world separated from school as a consequence of the closure of schools related to the pandemic. As a consequence, distance education was adopted. This may have an effect on the students negatively in different ways: less time spent on learning, development of depressive symptoms, change in the way of interaction, and lack of motivation for learning [7, 8].

Despite decisions to re-open schools, there are several strategies in Ethiopia to decrease person-to-person transmission of the pandemic among students. This includes decreased contact hours with teachers and encouragement of digital learning. This strategy may negatively affect students through the inaccessibility to information and communication technologies [9].

A Study found some predictors of adherence to communities for COVID-19 outbreak prevention measures. This includes self-efficacy, perceived benefits, perceived barriers, and perceived susceptibility [10].

The World health organization adopted a one-year strategic plan called COVID-19 strategic preparedness and response plan (SPRP) to be applied from February 1 2021 to January 31 2022 which is intended to help direct the public health response to the pandemic at different phases and to update the worldwide strategic priorities to support this response [11].

Several studies have been undertaken to measure COVID-19 knowledge, attitude, and practice. These studies, on the other hand, looked at a specific group of people, such as communities, health care providers, chronic disease patients, university students, and confined people. Only a few studies have examined the elements that can influence COVID-19 prevention. The purpose of this study was to determine the level of COVID-19 knowledge, attitude, preventative practice, and determinant factors among preparatory school students in Mizan-Aman town, Bench-Sheko zone.

## Methods and materials

### Study area and period

The study was conducted at the Mizan-Aman town secondary and preparatory schools. Mizan-Aman Town, the administrative center of the Benchi-Sheko zone, is located 574 km from Addis Ababa. It is located 227 km southwest of Jimma. There were three preparatory schools in the town. They provided services for a total of 2803 students, of them 1413 were male and 1390 were female. The data were collected from March 5 2021 to March 14, 2021.

### Study design

A cross-sectional study design was used.

### Study participants

All students registered in the Mizan-Aman town preparatory schools were the source population. Randomly selected students who fulfilled the inclusion criteria and were available during the data collection period were included in this study.

### Study variables

#### Dependent variables

Knowledge, Attitude, Practice.

#### Independent variables

Socio-demographic characteristics (age, gender, educational level, marital status), watching television, listening to the radio, having a cellphone, using social media, Knowledge, and attitude.

### Eligibility criteria

All students registered at Mizan-Aman town preparatory schools that were available during the data collection period were included in this study. Students who were unable to respond due to severe illness (fainting, coma, seizure episode) were excluded.

### Sample size determination and sampling technique

The sample size for this study was calculated by using single population proportion formula. Hence study has not been conducted on the KAP of COVID-19 among preparatory students, we used a prevalence of 50% to obtain the largest possible sample size. Considering a 95% confidence interval and a 5% margin of error, the total sample size became 384. By adding a 10% non-response rate, the final sample size was 422.

### Data collection instruments and procedure

Data were collected using a structured self-administered questionnaire. Information regarding sociodemographic data was collected using a structured self-administered questionnaire that was adopted after reviewing different kinds of literature [12]. The questionnaire was reviewed by research and community service experts. No major revision has been made on the tool. KAP towards COVID-19 prevention was assessed by using a students’ KAP on prevention of COVID-19 (SKAPCOV-19) questionnaire. The tool contains 36 items: 18 Knowledge items, 6 attitude items, and 12 practice items.

### Operational definitions

#### Preparatory school

An academic institution providing teaching-learning services for grade 11 and 12 students.

#### Knowledge

Those students who answered greater than or equal to the mean value of knowledge questions (≥9 questions) by the SKAPCOV-19 questionnaire were considered as knowledgeable [13].

#### Attitude

Those students who answered greater than or equal to the mean value of attitude questions (≥3 questions) by the SKAPCOV-19 questionnaire were considered as having a positive attitude [13].

#### Practice

Those students who answered greater than or equal to the mean value of practice questions (≥6 questions) by the SKAPCOV-19 questionnaire were considered as having good practice [13].

### Data quality assurance

Filled questionnaires were checked daily for errors and incompleteness. The data were collected by 4 data collectors (2 BSc Nursing and 2 BSc Public health). A two-day training was given for data collectors. Appropriate and pertinent instructions and directions were given for the data collectors to ensure the quality of data. The data collectors were supervised by an epidemiologist having MPH.

### Data processing and analysis

The collected data were checked for completeness and errors. Each questionnaire was coded and the data was entered and analyzed by using SPSS software version 25.0. The entered data were cleaned and errors were corrected. Hosmer-Lemeshow goodness of fit test was checked for model fitness and to check the fulfillment of the assumption of binary logistic regression. Multivariable binary logistic regression analysis was done to check the association of explanatory variables with COVID-19 prevention practice. Variables with a P-value of ≤ 0.05 in the multivariable binary logistic regression were considered a statistically significant determinant factor. Finally, the result of the study was summarized by using tables, graphs, and narrative descriptions.

### Ethical consideration

Ethical clearance was taken from the Ethical Review Committee of Mizan-Tepi University College of medicine and health science, Mizan-Aman town. Permission was taken from school directors to conduct the study. The respondents were informed about the objective of the study and the confidentiality of the information would be kept. Voluntary written informed consent was taken from the participants and verbal consent was taken from the parents of students under the age of18 years through phone calls. The participants were also informed that there were no financial benefits and harms from being involved in the study. They were informed about the right to discontinue the study at any time.

## Results

### Socio-demographic status of study participants

Out of the calculated 422 sample size, 406 have participated that yields a response rate of 96.2%. Among them 219(53.9%) were females and 187(46.1%) were males. The mean ages of the participants were 18.81±1.301 yrs. More than one-third, 177(43.6%), of the participants were Bench in ethnicity. Nearly half of the participants, 192(47.3%) were protestant in religion. The majority of participants, 340(83.7%), were unmarried (**Table 1**).

**Table 1:** Socio-demographic status of preparatory school students at Mizan-Aman town, 2021 (n = 406).

Variables	Category	Frequency	Percent
Age (mean ± SD)	18.81±1.301 years		
Gender	Male	187	46.1
	Female	219	53.9
Ethnicity	Bench	177	43.6
	Amhara	112	27.6
	Kaffa	56	13.8
	Oromo	24	5.9
	Others*	37	9.1
Grade level	Grade 11	283	69.7
	Grade 12	123	30.3
Religion	Protestant	192	47.3
	Muslim	64	15.8
	Orthodox	120	29.6
	Others**	30	7.4
Marital status	Single	340	83.7
	Married	66	16.3
Watching television	No	102	25.1
	Yes	304	74.9
Listening radio	No	166	40.9
	Yes	240	59.1
Having cellphone	No	62	15.3
	Yes	344	84.7
Social media users	No	142	35.0
	Yes	264	65.0

* **Tigray, Gurage, Silte, Sheka.**

** **Chatolic, Waqe feta.**

### Knowledge status of study participants about COVID-19

This study revealed that more than three-quarters of the participants, 332 [81.8%, 95% CI: 78–86%], were knowledgeable about COVID-19. Seventy two (18.2%) were not knowledgeable towards COVID-19.

Majority of the participants, 85.2%, knew that COVID-19 is caused by virus and 89.9% knew that fever and dry cough are the main clinical symptoms (**Table 2**).

**Table 2:** Response to knowledge questions among preparatory school students at Mizan-Aman town, 2021 (n = 406).

Questions	Responses	
	Yes N (%)	No N (%)
COVID-19 is caused by virus	346 (85.2)	60 (14.8)
The main clinical symptoms of COVID-19 are fever and dry cough	365 (89.9)	41 (10.1)
COVID-19 may also show no symptoms	189 (46.6)	217 (53.4)
Senior citizens aged 65 and older are at increased risk for COVID-19	295 (72.7)	111 (27.3)
People with chronic disease or co-morbid conditions are at increased risk	297 (73.2)	109 (26.8)
Children and teenagers are at the lowest risk for COVID-19	238 (58.6)	168 (41.4)
People having weak immune system are at increased risk for COVID-19	284 (70.0)	122 (30.0)
COVID-19 can be spread by an infected person without symptoms	314 (77.3)	92 (22.7)
COVID-19 can be spread by respiratory droplets of an infected person	327 (80.5)	79 (19.5)
COVID-19 can be spread by the dead bodies of the infected person	271 (66.7)	135 (33.3)
COVID-19 can be spread by the buried dead bodies of the infected person	200 (49.3)	206 (50.7)
COVID-19 cannot penetrate cloth masks	256 (63.1)	150 (36.9)
COVID-19 spreads by objects other than airborne	278 (68.5)	128 (31.5)
There is no effective drug for COVID-19	314 (77.3)	92 (22.7)
Avoid going to crowded place can prevent contracting COVID-19	359 (88.4)	47 (11.6)
Avoid traveling across cities can prevent contracting COVID-19	261 (64.3)	145 (35.7)
Not touching the face can prevent contracting COVID-19	313 (77.1)	93 (22.9)
Isolation of infected person can prevent contracting COVID-19	315 (77.6)	91 (22.4)

### Attitude status of the study participants towards COVID-19

In this study, more than two-thirds, 288 [70.9%, 95% CI: 67–75%], of study participants had a positive attitude towards COVID-19. One hundred eighteen (29.1%) of the participants had negative attitude towards COVID-19.

More than half, 63.3%, of the participants agreed that handling COVID-19 will be difficult if people or the community not keeping up with the information related to preventions (**Table 3**).

**Table 3:** Response to attitude questions among preparatory school students at Mizan-Aman town, 2021 (n = 406).

Questions	Responses		
	Agree N(%)	Do not know	Disagree
Handling COVID-19 will be more difficult if people or the community not keeping up with the information related to preventions	257 (63.3)	65 (16.0)	84 (20.7)
Handling COVID-19 will be more difficult if people or the community no longer need to worry about contracting COVID-19	268 (66.0)	86 (21.2)	52 (12.8)
Handling COVID-19 will be more difficult if people or the community influence by negative news	246 (60.6)	82 (20.2)	78 (19.2)
I feel that the person experiencing the symptoms or person infected should compliance the health protocol such as wearing the mask	279 (68.7)	91 (22.4)	36 (8.9)
I feel that person experiencing the symptoms or person infected should isolate themselves during 14 days	264 (65.0)	114 (28.1)	28 (6.9)
I feel that person experiencing the symptoms or person infected should be motivated to increasingly implement COVID-19 prevention measures and ensuring a healthy life	224 (55.2)	113 (27.8)	69 (17.0)

### COVID-19 prevention practice of the study participants

This study showed that nearly half of the participants, 191 [47%, 95% CI: 42–52%], have good practice and 215 (53.0%) of the participants have poor practice towards preventive measures of COVID-19.

About 70.7% of the participants responded that they sometimes wear a Mask. Only 66(16.3%) of the respondents often keep physical distancing (**Table 4**).

**Table 4:** Response to practice questions among preparatory school students at Mizan-Aman town, 2021 (n = 406).

Questions	Responses		
	Never N(%)	Sometimes N(%)	Often N(%)
Do you wear a mask in a crowded or public place	62 (15.3)	287 (70.7)	57 (14.0)
Do you keep a distance (physical distance) in a crowded or public place	137 (33.7)	203 (50.0)	66 (16.3)
Do you use hand sanitizer in a crowded or public place	175 (43.1)	165 (40.6)	66 (16.3)
Do you wash your hand and take a bath after going to a crowded or public place	138 (34.0)	138 (34.0)	130 (32.0)
Do you change your clothes after going to a crowded or public	174 (42.9)	138 (34.0)	94 (23.1)
Do you carry out a campaign to prevent the spread of COVID-19 through social media	137 (33.7)	187 (46.1)	82 (20.2)
Do you carry out a campaign to prevent the spread of COVID-19 by providing a direct example in daily activity	130 (32.0)	200 (49.3)	76 (18.7)
Do you eat fruits and vegetables in the last few days?	48 (11.8)	212 (52.2)	146 (36.0)
Do you get enough rest in the last few days?	92 (22.7)	207 (51.0)	107 (26.3)
In the last few days, do you exercise routinely?	124 (30.6)	180 (44.3)	102 (25.1)
In the last few days, do you take vitamins and supplements	174 (42.9)	162 (39.9)	70 (17.2)
Do you clean your house more frequently	57 (14.1)	165 (40.6)	184 (45.3)

### Factors associated with COVID-19 prevention practice

Concerning this study, those who were watching television implemented COVID-19 prevention measures [AOR: 1.884, 95% CI: (1.093, 3.247)] than those who were not watching television. Students who were social media (Facebook, telegram) users applied COVID-19 prevention measures [AOR: 1.801 95% CI: (1.005, 3.226)] than those who were not social media users. Students with good knowledge applied the COVID-19 prevention measures [AOR: 5.173 95% CI: (2.276, 11.755)] than those who were not knowledgeable. Students with a positive attitude were more likely to apply the COVID-19 prevention measures [AOR: 4.3 95% CI: (2.351, 7.868)] than those with a negative attitude towards COVID-19 **(Table 5)**.

**Table 5:** Factors associated with COVID-19 prevention practice among preparatory school students at Mizan-Aman town, 2021 (n = 406).

Variables	Category	Practice		COR (95% CI)	P-value	AOR (95% CI)	P- value
		Good	Poor				
Age	< = 18	88	98	1		1	
	>18	103	117	0.980(.663, 1.450)	0.921	1.626(.968, 2.732)	0.066
Gender	Male	82	105	1		1	
	Female	109	110	1.269(.857, 1.878)	0.234	1.315(.803, 2.153)	0.276
Grade level	Grade 11	141	142	1		1	
	Grade 12	50	73	0.690(.449, 1.059)	0.090	.601(.348, 1.037)	0.067
Marital status	Single	158	182	1		1	
	Married	33	33	1.152(.680, 1.952)	0.599	1.082(.566, 2.069)	0.811
Watching television	No	30	72	1		1	
	Yes	161	143	2.702(1.669, 4.375)	< .001	1.884(1.093, 3.247)	0.023*
Listening radio	No	58	108	1		1	
	Yes	133	107	2.351(1.539, 3.481)	< .001	1.311(.803, 2.141)	0.278
Having cellphone	No	22	40	1		1	
	Yes	169	175	1.756(1.001, 3.079)	0.049	1.149(.549, 2.407)	0.712
Social media users	No	49	93	1		1	
	Yes	142	122	2.209(1.448, 3.370)	< .001	1.801(1.005, 3.226)	0.048*
Knowledge status	Not Knowledgeable	8	66	1		1	
	knowledgeable	183	149	10.133(4.715, 21.774)	< .001	5.173(2.276, 11.755)	< .001*
Attitude status	Negative	21	97	1		1	
	Positive	170	118	6.655(3.929, 11.271)	< .001	4.300(2.351, 7.868)	< .001*

## Discussion

According to this study, 81.8% of the students were knowledgeable towards COVID-19 which is in line with a cross-sectional study done in China undergraduate students and Cameroonian in which 82.34%, and 84.19% of the participants were knowledgeable towards COVID-19 respectively [14, 15]. The result of this study is lower as compared with a study conducted in China and Ataye Hospital visitors in which 90% and 95.1% of the participants were knowledgeable towards COVID-19 respectively [16, 17]. This may be due to a difference in source population and difference in sample size as such factors may influence findings. Whereas, the result of this study is higher as compared with a study conducted at Gondar city secondary school students [18], on Bangladesh residents, a study conducted among undergraduate students at Debre Birhan University, and a study conducted on quarantined individuals in Tigray which showed that 23.5%, 48.3%, 73.8%, and 42.9% of the participants had accurate knowledge towards COVID-19 respectively [19–21]. It is also higher as compared with a study done on Jimma University medical center visitors which showed that 41.3% of the participants had a high knowledge level towards COVID-19 [22]. This difference may be due to a difference in the time at which the studies were conducted. The level of knowledge may be higher in recent studies due to disseminated information regarding the outbreak as it is a great public and government concern.

According to this study, 70.9% of the respondents had a positive attitude towards COVID-19, which is similar to a study conducted on Vietnamese [23] and with a report from Ethiopia [24] in which 68.6% and 72.9% of the participants had a positive attitude towards COVID-19 respectively. The result of the study is lower as compared with a study conducted in Malaysia which showed that 83.1% of the respondents had a positive attitude towards COVID-19 [25]. This difference may be due to a variation in the source population on which the study was conducted. However, the result of this study is higher as compared with a study conducted in Uganda which showed that 51.3% of the participants had a positive attitude towards COVID-19 [26]. It is also higher than a study conducted in Sidama regional state that showed 37.5% of the respondents had a positive attitude towards COVID-19 [27]. This may be due to a difference in the time at which the studies were conducted. An expanded health education program by the government and other non-governmental organizations may trigger the development of a positive attitude by the population.

According to this study, 47.0% of the respondents had good practice of COVID-19 prevention which is similar to studies conducted at Addis Zemen Hospital of Northwest Ethiopia and among Dessie city residents in which the prevalence of good practice was 52.7% and 44.6% respectively [13, 28]. The level of COVID-19 prevention practice by this study is slightly lower than those studies conducted among Dessie health center visitors and Amhara region health care workers in which 58.3% and 62% of the participants had good COVID-19 prevention practice [29, 30]. The level of practice determined by this study is markedly lower as compared with a survey conducted on undergraduate students in China in which 87.94% of the respondents had a proactive COVID-19 prevention practice [14]. This may be due to a difference in geographical location and sample size. As the outbreak was originated in China, several interventions and strategies by the government of China may influence the COVID-19 prevention practice. In another way, a difference in sample size affects the result of a study in which the larger sample size results in a more accurate determination of findings. The finding of this study is higher as compared with a study conducted at Oromia Special zone surrounding Finfinee [31] and on adult populations of Sidama regional state [27] in which 31.0% and 24.4% of the respondents adhered to COVID-19 prevention measures respectively. This may be due to a difference in source population in which students may be more likely to apply COVID-19 prevention measures as schools commenced rules and regulations regarding COVID-19.

According to this study, watching television has a statistically significant association with COVID-19 prevention practice in which students watching television were more likely to practice COVID-19 prevention measures. Several messages regarding COVID-19 are being broadcasted through different television channels and it will serve as one way of creating awareness towards the outbreak. Such messages may trigger the population to practice COVID-19 prevention measures.

Students who were social media users were more likely to practice COVID-19 prevention measures. This is similar with a study conducted in Jordan in which only small proportion of adolescents were engaged in risky practice while majority of them having information through social media and Television [32]. As it is known, social media have a great impact to alter the community towards a certain behavior. Different government and private social media channels disseminate messages towards the precaution of the COVID-19 outbreak.

According to this study, knowledge towards COVID-19 has a statistically significant association for prevention practice in which respondents who were knowledgeable regarding COVID-19 were more likely to practice COVID-19 prevention measures which is similar to a study conducted on Vietnamese and on Ataye Hospital visitors [17, 23]. The finding is also similar to a study conducted on quarantined patients in Tigray in which knowledgeable individuals were more likely to avoid traveling to crowded places, to wear a mask, and to apply strategies directed by the local health care authorities [21]. A study conducted on Jimma Medical center visitors also showed that respondents with good COVID-19 knowledge were more likely to wash hands frequently and to avoid handshaking [22]. This may be due to the effect of being knowledgeable towards the cause, transmission, and prevention of COVID-19 for the application of prevention measures. As someone becomes aware of the cause, transmission, and prevention of the disease, the more likely he/she apply COVID-19 prevention measures.

According to this study, attitude towards COVID-19 have a statistically significant association with COVID-19 prevention practice in which students having a positive attitude were more likely to have good COVID-19 prevention practice. The finding of this study is similar to a study conducted in China on undergraduate students which found a positive correlation between attitude and prevention practice of COVID-19 [14]. This may be due to the effect of positive feelings towards the prevention of COVID-19 for being adhered to prevention measures. As someone has felt positive towards prevention of the disease, the more likely he/she apply COVID-19 prevention measures.

### Implications of the study

The findings from this study provide baseline information regarding the KAP of preparatory students regarding COVID-19. As COVID-19 is the burning issue globally, the findings will guide policymakers, governmental and non-governmental organizations, to design strategies accordingly. It also directs the local health department to select the appropriate way for transmitting COVID prevention and precaution messages and to identify different constraints for the application of COVID prevention measures. The findings of this study will serve as a baseline for other researchers interested in related issues.

### Limitations of the study

Hence self-administered questionnaire was used, it is impossible to look for the feeling of the respondents while answering the questions. As a cross-sectional study assesses the causes and effects simultaneously, it may not show a perfect cause-effect relationship. On the other hand, the findings of this study may not be representative of other countries with different socioeconomic characteristics as a result of a difference in the degree of impact of the pandemic and may not be generalizable for a population with different socioeconomic classes.

## Conclusion

This study found that there was a high level of knowledge and a moderate level of a positive attitude toward COVID-19 among preparatory students. However, the practice of COVID-19 prevention measures was low.

Using social media and watching television increases the tendency of the students to practice COVID-19 prevention measures. Being knowledgeable and having positive attitudes towards COVID-19 by the students increases the chance for proper practice of COVID-19 prevention measures. The federal ministry of health and other governmental and non-governmental organizations should strengthen the transmission of messages concerning COVID-19 through social media and other national public Media. School directors and teachers should strictly monitor the students for their adherence to the COVID-19 prevention measures as directed by the local and national health care departments.

## Supporting information

S1 Data Minimal datasets: SPSS data. (SAV)

## Abbreviations

AOR: Adjusted Odds Ratio
CDC: Center for Disease prevention and Control
COR: Crude Odds Ratio
COVID-19: Coronavirus Disease 2019
KAP: Knowledge Attitude Practice
MERS: Middle East Respiratory Syndrome
SARS: Severe Acute Respiratory Syndrome
SKAPCOV-19: Students’ KAP on prevention of COVID-19
SPSS: Statistical Package for Social Science

## References

[1] Shereen MA, Khan S, Kazmi A, Bashir N, Siddique R. COVID-19 infection: Origin, transmission, and characteristics of human coronaviruses. J Adv Res. 2020;24:91–8. doi: 10.1016/j.jare.2020.03.005 32257431 PMC7113610

[2] European Centre for Disease Prevention and Control. Novel coronavirus disease 2019 (COVID-19) pandemic: increased transmission in the EU/EEA and the UK–sixth update. Stockholm; 2020.

[3] World Health Organization. Coronavirus Disease (Covid-19) Outbreak: Rights, Roles and Responsibilities of Health Workers, Including Key Considerations for Occupational Safety. Geneva; 2019.

[4] Ethiopian Public Health Institute. COVID-19 PANDEMIC PREPAREDNESS AND RESPONSE IN ETHIOPIA WEEKLY BULLETIN. 2021.

[5] World Health Organization. Corona virus—Sign and Symptom [Internet]. 2020 [cited 2021 Mar 20]. Available from: https://scholar.google.co.id/scholar?q=sign+and+symptom+of+infection&btnG=&hl=en&as_sdt=0%2C5&as_vis=1

[6] World Health Organization. Modes of transmission of virus causing COVID-19: implications for IPC precaution recommendations [Internet]. 2020 [cited 2020 Mar 20]. Available from: https://www.who.int/news-room/commentaries/detail/modes-of-transmission-of-virus-causing-covid-19-implications-for-ipc-precaution-recommendations

[7] Di Pietro G, Biagi F, Costa P, Karpiński Z, Mazza J. The Likely Impact of COVID-19 on Education: Reflections based on the Existing Literature and Recent International Datasets [Internet]. Publications Office of the European Union, Luxembourg. Luxembourg; 2020. Available from: www.stock.adobe.com

[8] Tadesse S, Muluye W. The Impact of COVID-19 Pandemic on Education System in Developing Countries: A Review. Open J Soc Sci. 2020;08(10):159–70.

[9] Espino-Díaz L, Fernandez-Caminero G, Hernandez-Lloret CM et al. Analyzing the impact of COVID-19 on education professionals. Toward a paradigm shift: ICT and neuroeducation as a binomial of action. Sustain. 2020;12:5646.

[10] Yehualashet SS, Asefa KK, Mekonnen AG et al. Predictors of adherence to COVID-19 prevention measure among communities in North Shoa Zone, Ethiopia based on health belief model: A cross-sectional study. PLoS One. 2021;16(1):1–16. 10.1371/journal.pone.0246006 PMC7822535 33481962

[11] World Health Organization. COVID-19 Strategic Preparedness and Response Plan. Geneva; 2021.

[12] Saefi M, Fauzi A, Kristiana E, Adi WC, Muchson M, Setiawan ME, et al. Validating of Knowledge, Attitudes, and Practices Questionnaire for Prevention of COVID-19 infections among Undergraduate Students: A RASCH and Factor Analysis. EURASIA J Math Sci Technol Educ. 2020;16(12):1305–8223.

[13] Alemu T, Amare S, Legesse S, Abera A, Ayalew M, Bezabih B. COVID-19 Knowledge, Attitude, Practices and Their Associated Factors Among Dessie City Residents, Northeast Ethiopia: A Cross-Sectional Study. Risk Manag Healthc Policy. 2021;14:439–51. doi: 10.2147/RMHP.S287600 33574719 PMC7871174

[14] Peng Y, Pei C, Zheng Y, Wang J, Zhang K, Zheng Z, et al. A cross-sectional survey of knowledge, attitude and practice associated with COVID-19 among undergraduate students in China. BMC Public Health. 2020;20(1292). doi: 10.1186/s12889-020-09392-z 32847554 PMC7447607

[15] Ngwewondo A, Nkengazong L, Ambe LA, Ebogo JT, Mba FM, Goni HO, et al. Knowledge, attitudes, practices of/towards COVID 19 preventive measures and symptoms: A cross-sectional study during the exponential rise of the outbreak in Cameroon. PLoS Negl Trop Dis. 2020;14(9):1–15. doi: 10.1371/journal.pntd.0008700 32886678 PMC7497983

[16] Zhong BL, Luo W, Li HM, Zhang QQ, Liu XG, Li WT, et al. Knowledge, attitudes, and practices towards COVID-19 among chinese residents during the rapid rise period of the COVID-19 outbreak: A quick online cross-sectional survey. Int J Biol Sci. 2020;16(10):1745–52. doi: 10.7150/ijbs.45221 32226294 PMC7098034

[17] Gebretsadik D, Ahmed N, Kebede E, Gebremicheal S, Belete MA, Adane M. Knowledge, attitude, practice towards COVID-19 pandemic and its prevalence among hospital visitors at Ataye district hospital, Northeast Ethiopia. PLoS One [Internet]. 2021;16(2):e0246154. Available from: http://www.ncbi.nlm.nih.gov/pubmed/33606678 doi: 10.1371/journal.pone.0246154 33606678 PMC7895377

[18] Handebo S, Adugna A, Kassie A, Shitu K. Determinants of COVID-19--related knowledge and preventive behaviours among students in reopened secondary sectional study schools: cross-sectional study. BMJ Open. 2021;(11):1–10. doi: 10.1136/bmjopen-2021-050189 33895723 PMC8076628

[19] Ferdous MZ, Islam MS, Sikder MT, Mosaddek ASM, Zegarra-Valdivia JA, Gozal D. Knowledge, attitude, and practice regarding COVID-19 outbreak in Bangladesh: An onlinebased cross-sectional study. PLoS One [Internet]. 2020;15(10 October):1–17. Available from: 10.1371/journal.pone.0239254 PMC7546509 33035219

[20] Aynalem YA, Akalu TY, Gebresellassie GB et al. Assessment of undergraduate student knowledge, attitude, and practices towards COVID-19 in Debre Berhan University, Ethiopia. PLoS One. 2021;16(5). 10.1371/journal.pone.0250444 PMC8130923 34003825

[21] Haftom M, Petrucka P, Gemechu K, Mamo H, Tsegay T, Amare E, et al. Knowledge, attitudes, and practices towards covid-19 pandemic among quarantined adults in Tigrai region, Ethiopia. Infect Drug Resist. 2020;13:3727–37. doi: 10.2147/IDR.S275744 33116693 PMC7585797

[22] Kebede Y, Yitayih Y, Birhanu Z, Mekonen S, Ambelu A. Knowledge, perceptions and preventive practices towards COVID-19 early in the outbreak among Jimma university medical center visitors, Southwest Ethiopia. PLoS One [Internet]. 2020;15(5):1–15. Available from: doi: 10.1371/journal.pone.0233744 32437432 PMC7241810

[23] Van Nhu H, Tuyet-Hanh TT, Van NTA, Linh TNQ, Tien TQ. Knowledge, Attitudes, and Practices of the Vietnamese as Key Factors in Controlling COVID-19. J Community Health [Internet]. 2020;45:1263–9. Available from: doi: 10.1007/s10900-020-00919-4 32894387 PMC7476242

[24] Yazew BG, Abate HK, Mekonnen CK. Knowledge, Attitude and Practice Towards COVID-19 in Ethiopia: A Systematic Review; 2020. Patient Prefer Adherence. 2021;15:337–48. doi: 10.2147/PPA.S288186 33623375 PMC7894797

[25] Azlan AA, Hamzah MR, Sern TJ, Ayub SH, Mohamad E. Public knowledge, attitudes and practices towards COVID-19: A cross-sectional study in Malaysia. PLoS One [Internet]. 2020;15(5):1–15. Available from: doi: 10.1371/journal.pone.0233668 32437434 PMC7241824

[26] Okello G, Izudi J, Teguzirigwa S, Kakinda A, Van Hal G. Findings of a Cross-Sectional Survey on Knowledge, Attitudes, and Practices about COVID-19 in Uganda: Implications for Public Health Prevention and Control Measures. Biomed Res Int. 2020;2020. 10.1155/2020/5917378 PMC7729389 34031643

[27] Yoseph A, Tamiso A, Ejeso A. Knowledge, attitudes, and practices related to COVID-19 pandemic among adult population in Sidama Regional State, Southern Ethiopia: A community based cross-sectional study. PLoS One [Internet]. 2021;16(1 January):1–19. Available from: 10.1371/journal.pone.0246283 PMC7846007 33513211

[28] Akalu Y, Ayelign B, Molla MD. Knowledge, attitude and practice towards covid-19 among chronic disease patients at addis zemen hospital, Northwest Ethiopia. Infect Drug Resist. 2020;13:1949–60. doi: 10.2147/IDR.S258736 32612371 PMC7322118

[29] Asemahagn MA. Factors determining the knowledge and prevention practice of healthcare workers towards COVID-19 in Amhara region, Ethiopia: A cross-sectional survey. Trop Med Health. 2020;48(1). doi: 10.1186/s41182-020-00254-3 32839649 PMC7438679

[30] Gebretsadik D, Gebremichael S, Belete MA. Knowledge, Attitude and Practice Toward COVID-19 Pandemic Among Population Visiting Dessie Health Center for COVID-19 Screening, Northeast Ethiopia. Infect Drug Resist. 2021;14:905–15. doi: 10.2147/IDR.S297047 33716509 PMC7944117

[31] Feyisa ZT. Factors limiting youths’ practice of preventive measures toward the outbreak of COVID-19 in Oromia special zone surrounding Finfinnee. PLoS One. 2021;16(3). doi: 10.1371/journal.pone.0248495 33720979 PMC7959380

[32] Dardas LA, Khalaf I, Nabolsi M, Nassar O. Developing an Understanding of Adolescents’ Knowledge, Attitudes, and Practices Toward COVID-19. J Sch Nurs. 2020;36(6):430–41. doi: 10.1177/1059840520957069 32990150

